# Mechanical Properties of Corner Joints Made of Honeycomb Panels with Double Arrow-Shaped Auxetic Cores

**DOI:** 10.3390/ma13184212

**Published:** 2020-09-22

**Authors:** Adam Majewski, Tomasz Krystofiak, Jerzy Smardzewski

**Affiliations:** 1 Department of Furniture Design, Faculty of Forestry and Wood Technology, Poznan University of Life Sciences, 60-637 Poznań, Poland; jerzy.smardzewski@up.poznan.pl; 2 Department of Wood Based Materials, Faculty of Forestry and Wood Technology, Poznan University of Life Sciences, 60-637 Poznań, Poland; tomasz.krystofiak@up.poznan.pl

**Keywords:** corner, joints, auxetic, honeycomb, stiffness, strength

## Abstract

The development of both light and strong wood-derived materials is an interesting research area, particularly in terms of usability in, e.g., furniture constructions. Honeycomb panels being current industry standard are relatively thick (32 mm and 40 mm), thus their attractiveness in designing furniture is limited. In a few studies, it has been shown that honeycomb panels with paper cores are characterized by unsatisfactory mechanical properties, especially when the composite thickness is less than 20 mm. From the literature, it is also evident that mechanical properties might be improved by introducing auxetic features into the core structure. Even though it is a concept with great potential, there are a few studies dealing with honeycomb panels with auxetic cores made of paper. Furthermore, there is no research on the corner joints made from such material. For this reason, the aim of the study was to test the bending behavior of the corner adhesive joints made of honeycomb panels with double arrow-shaped auxetic cores. Within the research, the core cell was adopted based on literature and preliminary studies, paper auxetic cores were produced by the use of the designed and 3d printed device, and joints stiffness and strength were calculated analytically based on the experiment results. Evaluated corner joints stiffness, both in compression and tension test, is greater for samples made of panels with designed auxetic cores. Surprisingly, in the analyzed range of elasticity, it was statistically proved that the values of joint stiffness coefficient K did not vary significantly between compared joints pairs.

## 1. Introduction

Crucial aspect in terms of use safety of construction with semirigid joints is the way that elements are assembled. The role of joints is to provide construction, e.g., like furniture, with high rigidity and strength [1]. Before the influence of materials and technologies of joints quality were widely described, the analytical calculations of global furniture stiffness had been formulated [2,3,4]. Mathematical models from previous decade relate to specific joints’ factors connected with numerical simulations of furniture rigidity [5,6,7,8]. Finite elements method (FEM), which has its origins in 1967, was interesting to use in studies on furniture joints rigidity undertaken by many authors [9,10,11,12,13,14,15,16,17]. Mechanical properties of corner joints for furniture purposes have been reported by many authors extensively. It is worth mentioning that vast majority of studies involve commonly used wood-derived materials, such as particleboard (PB) or medium density fiberboard (MDF) and additional items like fittings, adhesives, edge banding, or wooden add-ons. From the literature analysis, it is evident that in terms of corner joints rigidity, researchers used similar test methods and the following aspects were taken into account:

- Influence of material density as well as stiffness and rigidity of fasteners. It is exposed in the literature that values of linear modulus of elasticity (MOE and MPa) are greater for joints made of materials with bigger densities and homogenous, nonporous structure. There are studies on joint strength by which number of construction forms were tested i.e., structural-adhesive (double dowel or lamello), conventional metal fasteners (screws and confirmats), and more innovative products from companies like Blum, Hafele, and Titus plus [18,19,20,21,22,23,24,25,26,27,28,29,30,31,32];
- Effect of number of fasteners on joint stiffness, which is greater when the joint contact surface is bigger. This tendency is proportional to the number of used fasteners [10,33,34,35,36,37];
- Influence of distribution of fasteners. For instance, for double dowel joint the most effective dowel distribution equals 96 mm [38,39,40,41,42];
- Influence of fastener/joint geometry on joint stiffness. The increase in fastener length and diameter results in joint strength improvement, although increasing length is more effective [6,36,43,44]. Material change as well as different walls inclination angle was taken into consideration in corner dovetail joint test. Among examined samples including 5 different inclination angle values (75°, 78°, 81°, 84°, and 87°), four types of materials (birch, oak, pine, and MDF) and two adhesives, polyvinyl acetate (PVAc) and Desmodur-VTKA (D-VTKA), the best option was oak with 84° glued with D-VTKA [45];
- Presence, type, and adhesives application technique. In the literature, there are studies concerning diversity of adhesives, glue layer thickness, and gluing application methods. In general, the use of adhesives significantly improves joint strength [12,18,22,46,47,48]. Furthermore, better stiffness is a result of using silicon glue rather than PVAc [21];
- Effect of narrow surfaces finishing, use of edge banding, as well as its type and thickness. There are studies in the literature on the rigidity of furniture joints made of laminated PB and MDF. In addition, sides of samples were covered with: melamine edge band 0.4 mm thick, PVAc (0.4, 1, and 2 mm thick) ,and birch veneer (0.4, 1, and 2 mm thick) [49]. It was proved that the strongest joint was made of MDF with melamine edge banding. Average joint strength was about 17% and 18% better in comparison to the second-best variant (PB and PVAc, 0.4 mm) in compression and tension tests, respectively. Fathollahzadeh et al. [50] subjected different types of furniture cases (made of laminated MDF and raw MDF in versions both with and without elements edging) to cyclic loading. It was found that the strength of construction elements with edging is 1.8 times higher;
- The effect of a fastener grain orientation changes in relation to the grain direction of the specimen on the pulled-out joint strength. The stress distribution in model consisting of a dowel embedded in a maple wood sample was checked numerically. The change in moisture content from 10% to 18% was simulated, and the direction of the dowel fibers was orientated at 0°, 30°, 60°, and 90° in relation to the fiber direction of the sample. It was shown that the highest virtual pull-out strength was observed for the variant with 90° [51];
- The influence of the back panel assembly method on the strength of the corner joints. The presence of the back panel significantly improves the strength of the connection. In one of the works, the assembly methods have been assessed, where a back panel was mounted by screws to 18 mm thick elements made of: raw PB, veneer-coated PB, plywood (PLY), and MDF. Back panels were installed directly and through wooden (birch) add-ons. In each case, the use of wooden add-ons increased the strength of the joint as a result of the screws being firmly embedded in the material [52];
- The influence of the fasteners mounting force on the joint strength. The numerical calculations have shown that the application of maximum force moment while screwing a confirmat-type fastener into PB does not cause it to crack along the thread. On the other hand, the board is damaged due to the pressure of the confirmat head [5]. In another work, different hinges mounting plates and drawer sliders assembly methods were tested: directly to the panels by Euro screws, wood screws and plastic muff, and wood screws and a mounting plate. Different torque values for screwing fasteners were taken into account. Based on the results, it was found out that the most advantageous way is to use screws with a muff and a force moment value of 1.342 Nm [42];
- The effect of the guide holes diameter for screwed-in connectors on the joint strength. In order to increase the strength of joint, it is recommended to make guide sockets with diameters of 75–80% of the nominal diameter of the fastener [53,54,55,56].

In the light of presented literature overview, it must be stated that so far, there are only a few reports on mechanical properties of honeycomb panels with paper cores, although the idea of sandwich-like panels is generally known from 1960s [57]. However, some authors indicated their limits in the context of furniture industry utility [58,59,60]. They are mainly connected with relatively small MOE in comparison to standard materials for furniture production. Despite this fact, honeycomb panels remain tempting proposition as a more sustainable alternative for solid wood and research for their mechanical properties’ improvement has been intensified in the course of past years. The use of light furniture elements, but with satisfactory strength, effects in increased mobility and significantly reduces the weight of a package with elements making both transportation and assembly more convenient. It is important, especially in the context of the phenomenon of ageing societies. Smardzewski and others [61] tested the influence of ambient temperature changes and air relative humidity on the stiffness of joints made of honeycomb panels with PB frames. It has been shown that increasing the values of these parameters results in the deterioration of joint stiffness by 25% and strength by 40%. In Koreny's work [40], the analysis of the strength of honeycomb furniture joints was presented. Some of the fasteners for honeycombs that are available on the market were taken into account. It was shown that to achieve the best effect, fastener components should be glued in. After being embedded in the panel, their symmetrical axes should coincide. It was concluded that specified strength is acceptable for the broaden use of lightweight panels in furniture construction and remains consistent with their structural capabilities. Research should be continued, especially with regard to the shear strength of the embedded fasteners. The forementioned studies focus only on the hexagonal shape of the core cells and involve fasteners. From the literature, it is evident that mechanical properties of honeycomb panel may be modified by changing the size and height of a core cell, hence their relative density [62,63]. Therefore, more attention should be put to the research aimed at furniture honeycomb panel elastic properties improvement by changing these features on the basis of the auxetic materials concept, ergo with negative Poisson's ratio [64,65,66]. Numerous aspects of the auxetic structures application as well as their elastic constants have been already described in the literature [67,68,69,70,71,72,73,74]. However, there are a few studies on furniture honeycomb panels with auxetic core cells. Smardzewski [75] compared strength and MOE of panels with paper cores with both hexagonal and re-entrant cells. The overall panels’ thickness was crucial, because unlike commonly known 32 or 40 mm thick composites, the research was based on 18 mm thickness. It was stated that panels with auxetic cores exposed increased mechanical properties, however honeycomb panels with two different orthotropy directions were analyzed. There is no information how the strength of panels differentiates when comparing results for samples with cores tested in both orthotropy directions. Some authors [76] also examined bending behavior of honeycomb panels with auxetic cores made of WoodEpox^®^. Elastic characteristic, as well as amount of dissipated energy were taken into consideration. Among tested layered panels, the most favorable mechanical properties were exposed by honeycombs with facings prepared from high-density fiberboard—HDF and PLY, although as comes about the relationship between the MOE or Modulus of Rupture (MOR, MPa) and the density of materials, the most effective was beam with PLY facings in longitudinal orthotropy direction. A wide spectrum of elastic constants was a result of the other research [77], in which honeycomb panels with lattice truss cores were tested. They were manufactured from biodegradable wood composite material—LayWood.

Summing up, based on a thorough literature review, it might be assumed that there are no studies regarding corner joints made of honeycomb panels with paper auxetic cores. Consequently, the potential use of such panels in the furniture construction has not been determined either, especially when their thickness is comparable to 18 mm PB or MDF. Bearing that in mind, it was decided to carry out research to determine the extent to which honeycomb panels with an auxetic cores affect the mechanical properties of a corner joint. Thus, the aim of the study was to evaluate the stiffness and strength of L-shaped joints incorporating both hexagonal (reference) and auxetic cores in joints compression and tension tests. To achieve results, it was necessary to firstly design auxetic core cell, develop production method of paper auxetic cores, prepare and test samples in terms of physical–mechanical properties of used materials, and then determine stiffness and strength of joints.

## 2. Materials and Methods

### 2.1. Materials

Materials used in the study were as follows: 2.5 mm thick HDF for facings; 0.18 mm thick craft paper for auxetic cores (grammage 118 g/m^2^), which was cut in 12 mm width strips; a band of reference paper honeycomb (0.18 mm thick and grammage of 120 g/m^2^) with hexagonal cell shape; a dispersion adhesive based on a vinyl acetate copolymer—Jowacoll 148.00 (Jowat SE, Detmold, Germany) for gluing paper strips into an auxetic double arrow-shaped core; 35 mm width particleboard frame slats calibrated to thickness of 11.7 mm for pressing panels sets; Racoll ECO 3 (Isar-Rakoll Chemie, Nienburg, Germany) adhesive based on PVAc for gluing panel sets consisting of facings (glue rate 100 g/m^2^), frames, and cores; 2 mm thick acrylonitrile-butadiene-styrene copolymer (ABS) (Fritz Egger GmbH & Co. OG, Weiberndorf, Austria) edge bands for 4 side covering narrow surfaces of joints elements; 1.75 mm polylactide (PLA) (3Dwydruki, Skórzewo, Poland) filament for 3D printing in Fused Deposition Modeling (FDM) technology for making the device used in auxetic core production process (3D printer–FlashForge Dreamer; FlashForge 3D Technology Co. Ltd., Jinhua, China); Jowat 2k SE Polymer 690.00 (Jowat SE, Detmold, Germany)—two-component silane-epoxide (SE) adhesive (glueline grammage 2.74 g) for L-shaped corner joints preparation for compression and tension tests (only glue, no fasteners).

### 2.2. Corner Joints Stiffness Calculation Method

**Figure 1:**
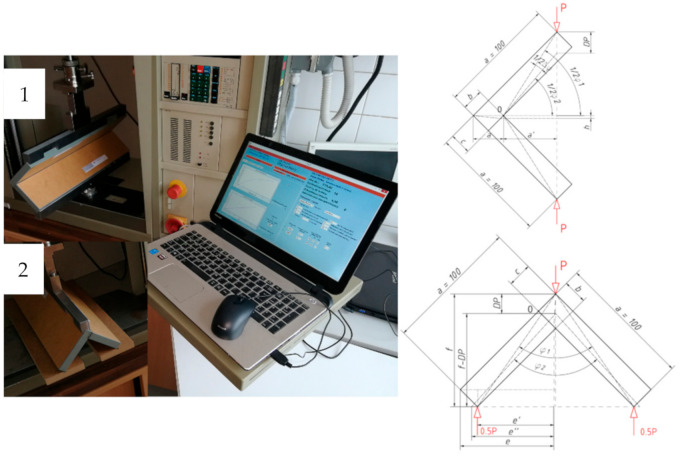
Zwick 1445 test machine and loading scheme for joint in (1) compression—C and (2) tension—T.

Two types of joint test were planned—compression (described as C) and tension (described as T). The calculation of the stiffness coefficient of corner joints was based on the method presented in the literature [44,78]. The advantage of this solution is the possibility to calculate analytically the value of the angle inclined between the elements instead of performing measurements using laboratory methods. Figure 1 illustrates the schemes on the basis of which equations were derived to determine the value of this angle in compressed and tensioned corner joints, as well as Zwick 1445 (Zwick Roell GmbH and Co.KG, Ulm, Germany) test machine with equipment used for the experiment.

Due to illustrated geometry, the stiffness coefficient K_c_ in compression test was calculated as follows:

(1) $$K_{c}=0.4P_{max}a^{\prime }/∆\varphi \;[\mathrm{Nm}/\mathrm{rad}]$$

(2) $$∆\varphi =\;\frac{\pi }{90}\left(\varphi_{1}-\varphi_{2}\right)$$

(3) $$a^{\prime }=\;\frac{\sqrt{2}}{2}a-a^{\prime \prime }$$

(4) $$a^{\prime \prime }=\;\sqrt{b^{2}+c^{2}-h^{2}}$$

(5) $$h=\frac{\sqrt{2}}{2}\left(b-c\right)$$

(6) $$\varphi_{1}=\mathrm{arctg}\left(\frac{\frac{\sqrt{2}}{2}a-h}{a^{\prime }}\right)$$

(7) $$\varphi_{2}=\arcsin\left(\frac{\frac{\sqrt{2}}{2}a-h-DP_{0.4P_{max}}}{\sqrt{c^{2}+{\left(a-b\right)}^{2}}}\right)$$

In the case of the corner joint tension test, the stiffness coefficient K_t_ equation was:

(8) $$K_{t}=0.4P_{max}e^{\prime \prime }/∆\varphi \;[\mathrm{Nm}/\mathrm{rad}]$$

(9) $$∆\varphi =\;\frac{\pi }{90}\left(\varphi_{2}-\varphi_{1}\right)$$

(10) $$e^{\prime }=\frac{\sqrt{2}}{2}\left(a-b\right)$$

(11) $$0.5\varphi_{1}=\mathrm{arctg}\left(\frac{e^{\prime }}{f}\right),$$

(12) $$0.5\varphi_{2}=\mathrm{arctg}\left(\frac{e^{\prime \prime }}{f-DP_{0.4P_{max}}}\right),$$

(13) $$f=e+\frac{\sqrt{2}}{2}b$$

(14) $$e^{\prime \prime }=\sqrt{e^{\prime 2}+f^{2}-{\left(f-DP_{0.4P_{max}}\right)}^{2}.}$$

The above equations, in addition to the parameters suitable for the test type, take into account the values of: destructive force P_max_ (N), the force is representing the conventional linear elastic limit P_0.4Pmax_ (N), and the corresponding displacement of DP_0.4Pmax_ (mm). They resulted from experiments performed on Zwick 1445 test machine setup as follows: test mode—bending with material destruction, speed of access to the material 10 mm/min, breaking force system 10 N, test speed 10 mm/min, force measurement accuracy 0.01 N, traverse displacement accuracy 0.01 mm, and maximum displacement set to 20 mm. The measurements were noted until the applied load drop of 50 N from the maximum force was observed. There was even load distribution to joint edge of 400 mm.

### 2.3. Corner Joints Rigidity Calculation Method

The strength of the corner joints has been calculated as the maximum bending moments of the joints. In accordance with the literature and Figure 1, it was considered as M_c_ = P_max_ * a’ (Nm) for compression and M_t_ = P_max_ * e’’ (Nm) for tension test.

## 3. Samples Preparation

To carry out the assumed research, it was necessary to: develop the shape of an auxetic core cell, select the shape of a reference hexagonal core cell, prepare paper cores with auxetic characteristic and reference ones, and produce honeycomb panels from which beams for elastic properties testing (without edge banding) and joints elements (elements covered four-sided with a 2 mm ABS edge) were prepared.

### 3.1. Core Cell Design

**Figure 2:**
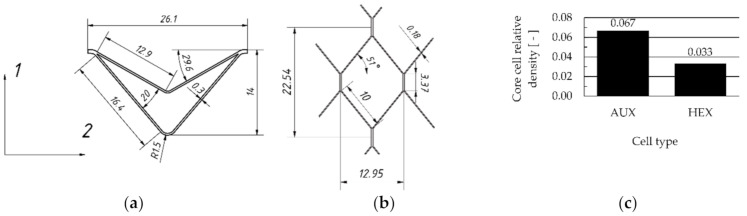
Tested core cells: (**a**) double arrow-shaped auxetic and (**b**) hexagonal and (**c**) relative density of cells.

Analytical descriptions of elastic properties of thin-walled cell structures with negative Poisson’s ratio are known in the literature [75,79,80,81]. The general shape of the auxetic core cell Figure 2a) has been selected based on the studies [70,82], but exact dimensions were adopted as a result of the author’s preliminary research, where elastic constants of a single cell were analyzed [17]. The shape of the reference cell was selected and measured (Figure 2b). The cell relative density was determined based on their 3D models made in Autodesk Inventor Professional 2018 software (Autodesk, Inc., San Rafael, CA, USA). The relative density of the auxetic cell was 0.067, whereas the relative density of a hexagonal one was 0.033 (Figure 2c).

### 3.2. Cores and Honeycombs Preparation

**Figure 3:**
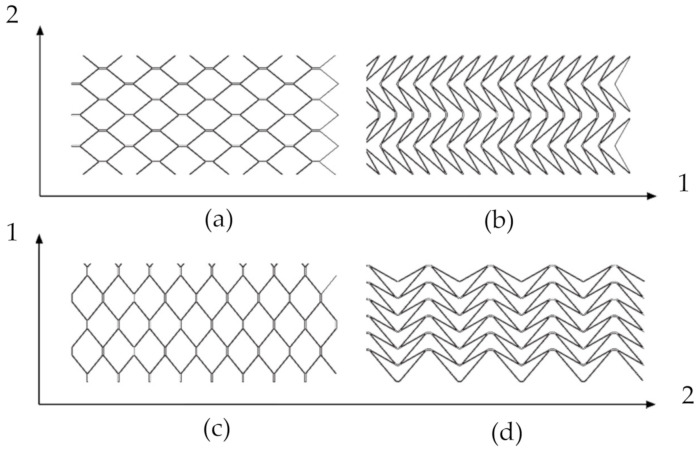
Orthotropy directions of panels: parallel for hexagonal HEX-1 (**a**) and auxetic cores AUX-1 (**b**) and perpendicular for hexagonal HEX-2 (**c**) and auxetic AUX-2 cores (**d**).

The description of cell arrangement in samples was adopted as HEX-1 and HEX-2, where HEX-1 and HEX-2 determines the direction of cell wall oriented parallel and perpendicular, respectively, to the longer side of the sample (Figure 3).

**Figure 4:**
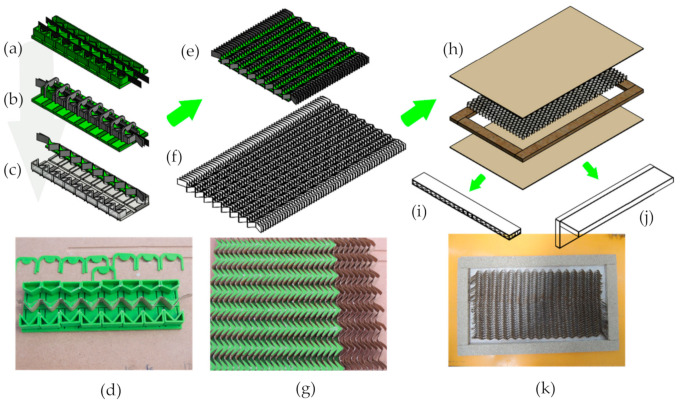
Honeycomb panels preparation method: paper strips were dragged along the designed and 3D printed device (**a**), then pressed successively by molds with simultaneous application of glue (**b**) to obtain a single slat with a row of core cells (**c**,**d**); then, 30 cell systems prepared in this way were glued in order to achieve the width of half of the assumed core dimension (**e**,**g**); after repeating the whole procedure, a paper core of the required dimensions of approximately 420 × 230 mm^2^ (**f**) was obtained; then the cores (auxetic and reference) were embedded in 11.7 mm (**k**) particleboard frames, covered with 2.5 mm HDF facings, and the sets (**h**) were pressed (p = 1 daN/cm^2^, t = 10 min, T = 20 °C); approximately 100 panels were produced in this way—with auxetic and hexagonal reference cores, from which beams for three-point bending (**i**) and elements of corner joints (**j**) were cut out.

The preparation method of honeycomb cores with auxetic cells is shown in Figure 4a,g, whereas the way of manufacturing honeycomb panels including both types of cores is shown in Figure 4h,k.

In total, 48 panels with auxetic cores, including both orthotropy directions were prepared. Dimensions of a single panel were 520 × 317 × 17 mm^3^.

### 3.3. Corner Joints Preparation

Cutting of the produced panels was carried out on the Felder K 700 Professional (Felder Group Polska Sp. z o.o., Żory, Poland) sawing machine. It was concluded that in order to stabilize the multilayered arrangement of the panels and to limit the delamination phenomenon in the early bending phase of the joints, the narrow surfaces of the samples should be covered on four sides with a 2 mm ABS edging. A HOMAG KFL 610 powerLine (HOMAG Group, Schopfloch, Germany) with Technomelt PUR 270/7 G (Henkel, Düsseldorf, Germany) polyurethane-based hot melt adhesive was used for this. Then, from the edge banded elements, the L-shaped joints intended for testing were made. It was crucial to prepare them in such a way that the contact surfaces of the elements could be considered as “perfectly rigid.” This way, the stiffness of the tested joints can be assessed in terms of the stiffness of the panels, not the stiffness of the used fastener. The undertaken technological tests showed that the best method to achieve this is to use Jowat 2k SE Polymer 690.00 for adhesive bonding. According to the manufacturer's recommendations, full curing of the joint requires about 7 days of seasoning in normal conditions (temperature 23 ± 2 °C and air humidity 50 ± 5%).

Eventually, bearing in mind the factors such as two test variants, two types of paper cores (auxetic and hexagonal), and two directions of core orthotropy, adequate samples were prepared, as shown in Table 1. Therefore, it was decided to use the ANOVA for further statistical analysis empowered by the Tukey’s honest significant difference (HSD) test for pairs of means comparison (in case of joints stiffness) and Student’s *t*-test for significance assessment of independent samples (in case of joints strength). Each corner joint consisted of two elements with dimensions: 400 × 83 × 17 mm^3^ and 400 × 100 × 17 mm^3^. The size of beams for bending was adopted according to standard [83]. It is worth mentioning that panels in HEX-2 core orientation are hardly present in industry practice.

## 4. Results and Discussion

### 4.1. Materials and Panels Properties

Mechanical properties of the paper used in the study in the machine (MD) and transverse (CD) orthotropy directions were determined in a tensile test in accordance with [84] using a Zwick 1445 machine and digital image analysis equipment (Digital Image Correlation—DIC). The measuring set consisted of a Dantec Dynamics KL50108 (Dantec Dynamics A/S, Skovlunde, Denmark) camera connected to a computer and Istra 4D 4.4.6.319 software. The MOE and MOR of HDF as well as manufactured multilayered panels were determined in accordance with [83]. For the HDF samples, the presence of adhesive located on the top (US) and bottom (LS) side of each sample was additionally considered. The moisture content of HDF facings [85], paper grammage [86], and paper thickness [87] were tested. The results of the physical–mechanical properties of materials and manufactured panels are recalled in Table 2 and Table 3, respectively. Their detailed analysis, including statistical tests, is presented in author’s other research [88]. As a result, panels thickness was as follows: AUX-1—17.05 mm (SD = 0.10 mm), AUX-2—17.07 mm (SD = 0.11 mm), HEX-1—17.17 mm (SD = 0.17 mm), and HEX 2—17.04 mm (SD = 0.04 mm).

Based on cited results, it can be seen that bigger MOE values had panels with cores in which common cell walls (glued together creating double-wall arrangement) were oriented perpendicular to the longer side of the sample. A similar tendency was expected to appear in terms of tested corner joints. Therefore, further results analysis was focused mainly on the comparison of pairs AUX-1: HEX-2 and AUX-2:HEX-1.

### 4.2. Corner Joints Stiffness

**Figure 5:**
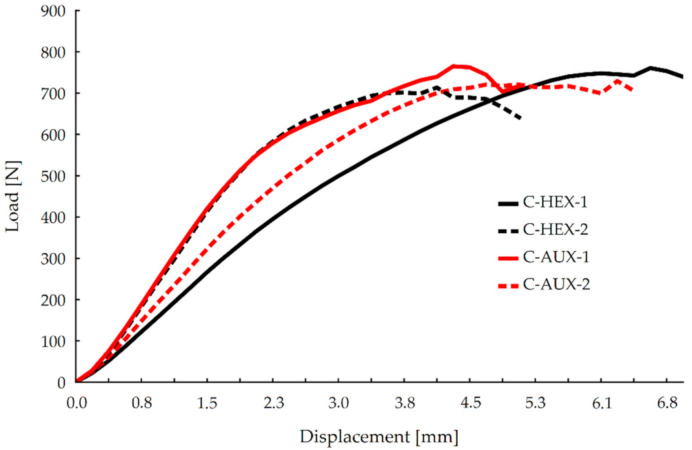
Stiffness of joints in the compression test.

It should be noted that the two-component SE adhesive Jowat 2k SE Polymer 690.00 used for joints preparation generated a durable and resilient bonding. This resulted in achieving relatively big maximum forces, which only caused the joint deflection without any visible damage of the top layers of bonded surfaces. Figure 5 shows the average stiffness of the corner joints in the compression test.

**Figure 6:**
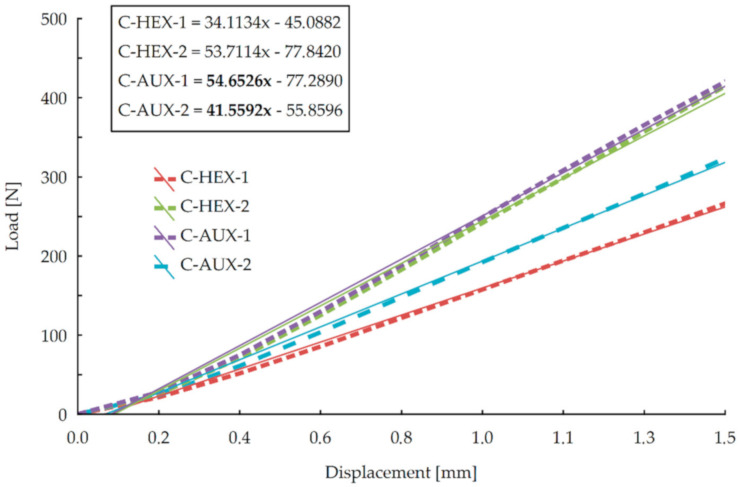
Stiffness of joints in compression test in the linear elasticity range.

It may be seen in this figure that all the compressed joints reached a maximum force of value 700 N and more. It can also be stated that stiffness of HEX-2 and AUX-1 nearly overlaps almost through the test, although after reaching deflection of 4 mm, AUX-1 joints gain bigger maximum force (about 800 N). In the displacement range of 2.3–4 mm, the average difference in the force value is 84.5 N. Relatively the biggest displacement increase with the smallest force increase was shown by HEX-1 type joints. In order to determine more precisely the stiffness of the joints in compression test, it was decided to focus on stiffness curves in the linear range of elasticity. It was found that for the value of the force equal to 40% of the destructive force P_max_, the limit of this range runs with a deflection of 1.5 mm. Then, for each joints type, linear regression was adjusted, and its equation was calculated, as shown in Figure 6.

**Figure 7:**
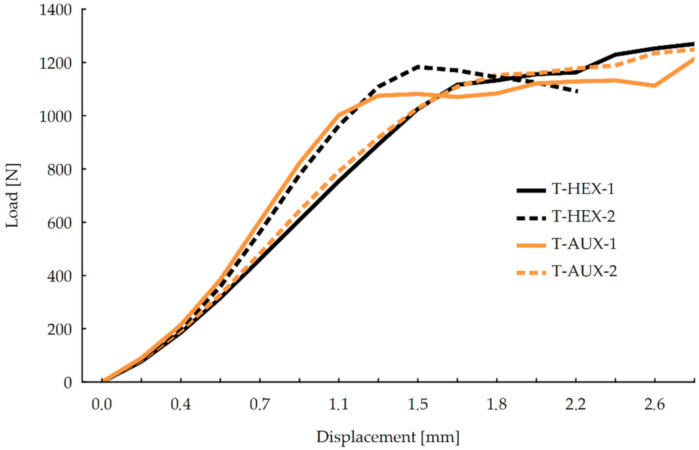
Stiffness of joints in the tension test.

On the basis of linear regression equations, it can be concluded that the directional coefficients of function have more significant values in the case of joints made of panels with auxetic cores, both in 1 and 2 orthotropic directions. There is a more significant difference between HEX-1 and AUX-2 joints than HEX-2 and AUX-1. Therefore, from this analysis, it follows that joints with auxetic cores exhibit greater rigidity in the compression test. An analogous evaluation was carried out for the results of joints in the tension test (T) (Figure 7).

**Figure 8:**
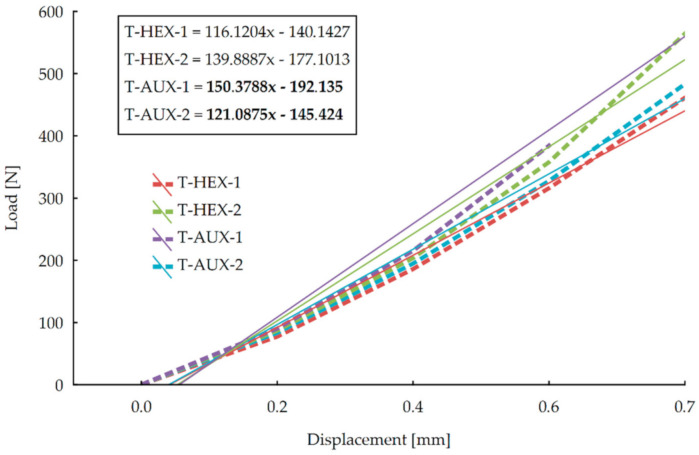
Stiffness of joints in tension test in the linear elasticity range.

It may be noticed that in the displacement range of 0–1.2 mm, the averaged stiffness curves of HEX-1 and AUX-2 as well as HEX-2 and AUX-1 joints have similar courses. In the case of HEX-1 and AUX-2, it is only from a displacement of approximately 2.2 mm that the curves differ—the HEX-1 shows a slightly higher average stiffness at the end of the test. For the HEX-2 and AUX-1 joints, the difference occurred at twice the displacement value, i.e., approximately 1.1 mm. In the 1.3–2 mm range, the stiffness of AUX-1 is slightly less than of HEX-2. In order to obtain a more complete description of the tensioned joints stiffness, it was decided to carry out an evaluation of matched linear regressions, as it was done in the evaluation of stiffness characteristics of the previous test, respectively. In this case, however, it was found that the range of linear elasticity is within the displacement range of 0–0.7 mm, as shown in Figure 8.

It is clear that similar to the compression test results, greater directional coefficients of regressions show the joints made of panels with auxetic cores. Thus, it can be concluded that in the case of tensioned joints, those with auxetic cores exhibit better stiffness for both directions of orthotropy.

**Figure 9:**
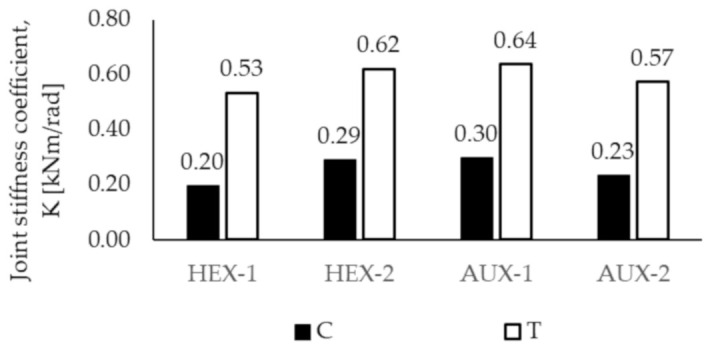
Stiffness coefficient K of corner joints in compression (C) and tension (T) test.

The above observations concerning the results of corner joints tests should be reflected in the determined stiffness coefficient K. Therefore, Figure 9 presents the calculated values.

It is evident that slightly bigger values of the stiffness coefficient, for both tests, resulted for corner joints made of panels with auxetic cores. The differences are as follows:

- For compression test: the stiffness coefficient of AUX-2 is 15% bigger than HEX-1 and AUX-1 is 3.4% bigger than HEX-2;
- For tension test: AUX-2 is 7.5% stiffer than HEX-1 and AUX-1 is 3.2% stiffer than HEX-2.

When evaluating the differences between the directions of core orthotropy in the samples, it can be observed that:

- The compressed AUX-1 joints expose a stiffness coefficient of 30.4% bigger than AUX-2, although the tensioned AUX-1 joints show a stiffness coefficient of 12.3% bigger than AUX-2;
- The compressed joints HEX-2 have a 45.0% bigger stiffness coefficient K than HEX-1, and tensioned HEX-2 show a stiffness coefficient of 17.0% bigger than HEX 1.

In addition, the stiffness coefficient K obtained for compressed joints is in the case of:

- HEX-1 by 165%,
- HEX-2 is 138%,
- AUX-1 by 133%,
- AUX-2 is 148% bigger than the value calculated for tensioned joints.

Then the obtained results had to be verified by statistical analysis of ANOVA variance for the factor systems. In the first step, a one-dimensional significance test for stiffness coefficient K was carried out (Table 4).

**Figure 10:**
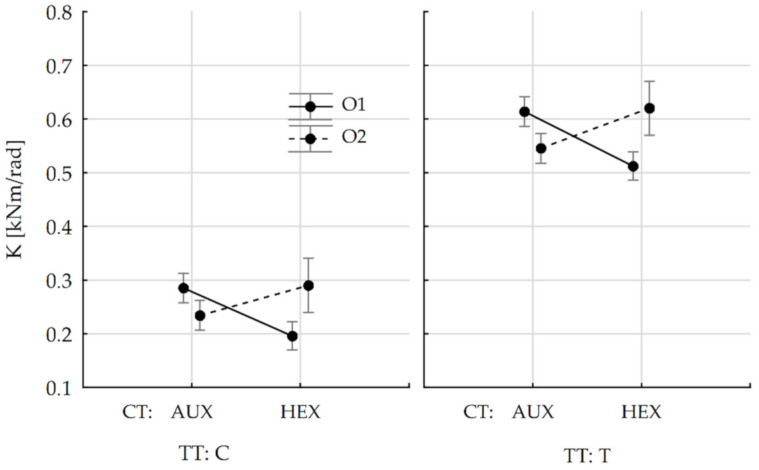
Change in stiffness coefficient depending on core type (CT) and orthotropy direction (O) and also type of joint test (TT) (vertical bars mean 0.95 confidence intervals).

Results presented in Table 3 indicate that among the effects taken into account, the stiffness of joints is influenced by the type of joint test and the interaction of the core type with the directions of orthotropy. Therefore, it was decided to trace these relations by determining the tendency of stiffness coefficient change for honeycomb panels joints with hexagonal (HEX) and auxetic (AUX) cores to the test type (TT) and orthotropic directions (O). The result is shown in Figure 10.

It can be seen that for the orthotropic direction 2, the K coefficient is bigger in both tests and joints with hexagonal cores show slightly bigger stiffness. For the orthotropic direction 1, the trend is reversed and the change is more marked in both tests in favor of honeycomb panels joints with the auxetic cores. It was, therefore, necessary to determine whether the results presented in the graph are statistically different. Tukey's HSD test was then carried out for the K variable (Table 5). All the statistical computations were performed with the Statistica 13.1 software (StatSoft Polska Sp. z o.o., Kraków, Poland).

Based on Tukey’s test’s results, it should be determined that the K-values of the joints pairs:

- In compression test: AUX-2:HEX-1 and AUX-1:HEX-2 do not differ significantly;
- In tension test: AUX-2:HEX-1 and AUX-1:HEX-2 do not differ significantly.

To sum up, the analysis of stiffness of the tested joints shows that in the range of linear elasticity, the linear regression curves for the ones with auxetic cores have bigger directional coefficients than the regressions of references with hexagonal cores. Stiffness coefficients of the former mentioned joints exposed bigger values, however, statistical analysis showed that these results do not differ significantly.

### 4.3. Corner Joints Strength

**Figure 11:**
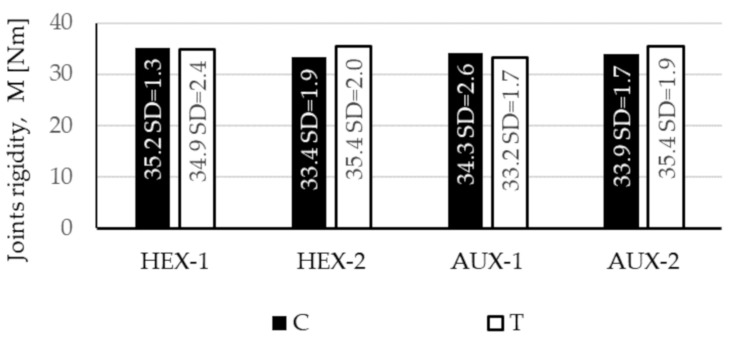
Rigidity of corner joints made of honeycomb panels in compression test (C) and tension test (T).

Calculated corner joints strength is presented in Figure 11.

The biggest strength of 35.4 Nm was shown by the tensioned AUX-2 (SD = 1.9 Nm) and HEX-2 (SD = 2.0 Nm) joints. This is 6.6% and 1.4% bigger than AUX-1 (Mt = 32.2 Nm, SD = 1.9 Nm) and HEX-1 (M_t_ = 34.9 Nm, SD = 2.4 Nm), respectively. The strength of the tensioned AUX-1 joints is at the same time the smallest of the obtained ones. The compression HEX-1 joints showed a strength of only 0.6% (M_c_ = 35.2 Nm, SD = 1.3 Nm) less than the maximum values. This result is at the same time 5.4% bigger than the strength of compressed HEX-2 joints (M_c_ = 33.4 Nm, SD = 1.9 Nm). The compressed AUX-2 strength of 33.9 Nm (SD = 1.7 Nm) is only 1.2% less than AUX-1 (Mc = 34.3 Nm, SD = 2.6 Nm). The joints strength seems to be very similar in both types of tests, as the biggest differences in values differ by several percentage points. Next, the Student’s statistical *t*-test for independent samples was performed to verify the significance of these differences at a level of *p* = 0.05. On the basis of the obtained results, it can be indicated that the values differ significantly when comparing the strength of joints: in compression test HEX-1 and AUX-2, compression HEX-1 and tension AUX-1, and tension AUX-1 and AUX-2.

## 5. Conclusions

On the basis of the research, the following conclusions and observations were drawn:

It was characteristic that greater stiffness coefficient in both compression and tension tests was achieved by panels with cores in which common cell walls were oriented perpendicular to longer sample side, namely, AUX-1 (0.3 kNm in the compression test and 0.64 kNm in tension test) and HEX-2 (0.29 kNm in compression and 0.62 kNm in tension test) comparing to AUX-2 (0.23 kNm in the compression test and 0.57 kNm in tension test) and HEX-1 (0.20 kNm in the compression test and 0.53 kNm in tension test). However, results of Tukey’s test showed that values for compared joints pairs HEX-1:AUX-2 and HEX-2:AUX-1 do not differ significantly. 

Between the compared pairs of joints, namely, AUX-2:HEX-1 and AUX-1:HEX-2, the average stiffness both in the compression and tension tests was slightly better in the case of panels with auxetic cores. Furthermore, in the range of linear elasticity, the directional coefficient of matched linear regressions showed a bigger value for joints made of panels with auxetic cores.

The use of double arrow-shaped auxetic cells, despite their double relative density compared to hexagonal reference cells, did not increase the modulus of linear elasticity. The mean MOE values of HEX-1 (2025 MPa, SD = 85 MPa) and AUX-2 (1852 MPa, SD = 47 MPa) panels differed significantly in favor of HEX-1, whereas for HEX-2 (1394 MPa, SD = 83 MPa) and AUX-1 (1381 MPa, SD = 83 MPa), the MOE values did not differ significantly. For this reason, cores with hexagonal cells remain attractive to be used for producing lightweight, layered furniture panels.

When it comes to the use of panels with auxetic cores, it is recommended to choose core orientation where glued common walls are arranged perpendicularly to the longer side of elements. These panels can be used in furniture structures, especially for horizontal partitions.

## Figures and Tables

**Table 1:** Summary of samples prepared for tests.

Test Type	Sample Code	Number of Samples	Sample Dimensions mm^3^
Beam 3-point bending	HEX-1	10	410 × 50 × 17
	HEX-2	10	410 × 50 × 17
	AUX-1	10	410 × 50 × 17
	AUX-2	10	410 × 50 × 17
Joint compression test	C-HEX-1	10	400 × 100 × 100
	C-HEX-2	5	400 × 100 × 100
	C-AUX-1	10	400 × 100 × 100
	C-AUX-2	10	400 × 100 × 100
Joint tension test	T-HEX-1	10	400 × 100 × 100
	T-HEX-2	5	400 × 100 × 100
	T-AUX-1	10	400 × 100 × 100
	T-AUX-2	10	400 × 100 × 100

**Table 2:** Physical and mechanical properties of materials used in the study [88].

Paper			HDF		
Property	MPa	SD	Property	MPa	SD
**MOE_MD_**	2236	389	**MOE_US_**	4017	150
**MOE_CD_**	692	156	**MOE_LS_**	3945	341
**MOR_MD_**	13	2	**MOR_US_**	42	2
**MOR_CD_**	5	1	**MOR_LS_**	39	4
**G_MD/CD_**	873	–	**G_US/LS_**	1762	-
**G_CD/MD_**	291	–			
	–			–	
**𝞾** **_MD/CD_**	0.28	0.07	𝞾**_US/LS_**	0.28	-
**𝞾** **_CD/MD_**	0.19	0.06			

**Table 3:** Physical and mechanical properties of tested honeycomb panels [88].

Property	MOE	MOR	ρ_r_
	MPa		kg/m^3^
**AUX-1**	1381	3.8	300.3
**SD**	83	0.2	4.1
**AUX-2**	1852	8	296.9
**SD**	47	0.8	3.5
**HEX-1**	2025	8.7	284.6
**SD**	85	0.4	5.1
**HEX-2**	1394	3.9	276.1
**SD**	83	0.3	2.2

**Table 4:** One-dimensional significance test for K coefficient (kNm/rad).

Effect	SS	Degrees	MS	F	p
**Free**	8,703,039	1	8,703,039	4,560,449	0,000,000
**TT**	1,326,762	1	1,326,762	695,232	0,000,000
**CT**	0,002,946	1	0,002,946	1544	0,218,916
**O**	0,005,477	1	0,005,477	2870	0,095,441
TT*CT	0,000,031	1	0,000,031	0016	0,899,017
TT*O	0,000,012	1	0,000,012	0006	0,937,948
CT*O	0,082,873	1	0,082,873	43,426	0,000,000
TT*CT*O	0,000,782	1	0,000,782	0410	0,524,591
**Error**	0,114,502	60	0,001,908		

**Table 5:** Tukey’s HSD test for K variable (Nm/rad).

			TT	C				T			
			CT	AUX		HEX		AUX		HEX	
	**TT**	**CT**	**O**	**1**	**2**	1	2	1	2	1	2
**1**	C	AUX	1	x	0.17299	0.00054	1.00000	0.00013	0.00013	0.00013	0.00013
**2**	C	AUX	2	0.17299	x	0.48487	0.52762	0.00013	0.00013	0.00013	0.00013
**3**	C	HEX	1	0.00054	0.48487	x	0.03181	0.00013	0.00013	0.00013	0.00013
**4**	C	HEX	2	1.00000	0.52762	0.03181	x	0.00013	0.00013	0.00013	0.00013
**5**	T	AUX	1	0.00013	0.00013	0.00013	0.00013	x	0.01850	0.00016	1.00000
**6**	T	AUX	2	0.00013	0.00013	0.00013	0.00013	0.01850	x	0.65786	0.17826
**7**	T	HEX	1	0.00013	0.00013	0.00013	0.00013	0.00016	0.65786	x	0.00794
**8**	T	HEX	2	0.00013	0.00013	0.00013	0.00013	1.00000	0.17826	0.00794	x
